# Phytochemical and Agronomic Characterization of High-Flavonoid Lettuce Lines Grown under Field Conditions

**DOI:** 10.3390/plants12193467

**Published:** 2023-10-02

**Authors:** Eunjin Cho, Csanad Gurdon, Rebecca Zhao, Hui Peng, Alexander Poulev, Ilya Raskin, Ivan Simko

**Affiliations:** 1 Department of Plant Biology, Rutgers University, New Brunswick, NJ 08901, USA; eunjin.cho@rutgers.edu (E.C.); csanadgurdon@gmail.com (C.G.); apoulev@rutgers.edu (A.P.); raskin@rutgers.edu (I.R.); 2 Crop Improvement and Protection Research Unit, US Department of Agriculture, Agricultural Research Service, Salinas, CA 93905, USA; rebecca.zhao@usda.gov; 3 Everglades Research and Education Center–Horticultural Sciences Department, University of Florida, Belle Glade, FL 33430, USA; huipeng@ufl.edu

**Keywords:** lettuce, flavonoids, anthocyanin, quercetin, kaempferol, naringenin chalcone, yield, chlorophyll, postharvest quality, field trial

## Abstract

Flavonoids are antioxidant phytochemicals that confer a beneficial effect on human health. We have previously developed and characterized eight lettuce (*Latuca sativa* L.) lines that accumulated high levels of diverse flavonoids and their precursors in controlled environment conditions. Three Rutgers Scarlet lettuce (RSL) lines selected in tissue culture for deep-red color (RSL-NAR, RSL-NBR, RSL-NFR) accumulate anthocyanins and quercetin, three lines identified in a chemically mutagenized red lettuce population accumulate kaempferol (KfoA and KfoB) or naringenin chalcone (Nco), and two lines that were spontaneous green mutants derived from the red line RSL-NAR (GSL, GSL-DG) accumulate quercetin. These eight lines were field-grown in the Salinas Valley of California for four years together with seven control accessions of varying colors (light green, dark green, red, and dark red). At market maturity, a substantial variation in plant composition was observed, but the three RSL lines consistently accumulated high levels of cyanidin, GSL and GSL-DG accumulated the highest levels of quercetin, KfoA and KfoB accumulated kaempferol, and Nco amassed naringenin chalcone, confirming that these mutant lines produce high levels of beneficial phytochemicals under field conditions. Mutant lines and control accessions were also assessed for their biomass production (plant weight, height, and width), overall content of pigments (leaf chlorophyll and anthocyanins), resistance to diseases (downy mildew, lettuce drop, and Impatiens necrotic spot virus), postharvest quality of processed tissue (deterioration and enzymatic discoloration), and composition of 23 mineral elements. All but one mutant line had a fresh plant weight at harvest comparable to commercial leaf cultivars; only Nco plants were significantly (*p* < 0.05) smaller. Therefore, except for Nco, the new, flavonoid hyperaccumulating lines can be considered for field cultivation.

## 1. Introduction

According to the World Health Organization, 17.9 million deaths, or 32% of all global deaths, in 2019 were caused by preventable issues surrounding cardiovascular diseases related to unhealthy diet and obesity [1]. People who consume a diet rich in fruits or vegetables have a reduced risk of chronic metabolic diseases such as obesity, cardiovascular disease, and diabetes [2,3]. Evidence shows that consumption of fruit and vegetable flavonoids produces health benefits; supported by studies conducted with several plant flavonoids. Green tea extracts have been found to reduce body fat, blood pressure, and low-density lipoprotein (LDL) cholesterol, all associated with a lower risk of cardiovascular disease [4]. Green tea epicatechins have shown to increase plasma antioxidant capacity and activity of antioxidant enzymes [5]. Quercetin and quercetin monoglucosides from onions, tomatoes, and apples were associated with significant reductions in deaths due to cardiovascular diseases in epidemiological studies. In addition, quercetin also inhibits 15-lipoxygenase, an enzyme that modifies LDL cholesterol and is associated with atherosclerosis development [6,7].

Lettuce (*Latuca sativa* L.) is the third-most commonly consumed vegetable in the US, with lettuce production contributing almost a fifth of all vegetable cash receipts in 2022; $1.54 billion coming from romaine, $1.33 billion from iceberg, and $1.25 billion from leaf lettuce [8]. Though lettuce breeding programs focus primarily on developing cultivars resistant to pests, pathogens, and physiological disorders, while providing high yield and long shelf life [9,10,11,12], recent efforts aimed to develop breeding lines with high levels of certain phytochemicals [13,14,15,16,17]. Lettuce is a good source of fiber, folic acid, vitamin C, iron, and carotenoids, whereas it displays a substantial variation among cultivars in the levels of phenolics, including phenolic acids and flavonoids [18,19]. The most abundant lettuce phenolic acids are derivatives of caffeic acid, and include chlorogenic, chicoric, caffeoyltartaric, and caffeoylmalic acids, while the most abundant flavonoids are flavonol glycosides, typically quercetin 3-O-malonylglucoside, quercetin 3-O-glucoside, and quercetin 3-O-glucuronide [20,21]. Additionally, red lettuces accumulate the anthocyanin cyanidin 3-O-malonylglucoside [20,21,22]. Phenolics and flavonoid accumulation depend on the lettuce type: low levels are found in crisphead (iceberg) cultivars, while red leaf and red oak lettuces contains the highest levels [18,21].

As the genetic potential for obtaining high levels of phenolics is present in the germplasm, development of novel lettuce cultivars with elevated phenolics levels is possible. Damerum et al. [14] crossed an accession of wild *Lactuca serriola* that contained high levels of phenolics with green iceberg type cv. Salinas containing low levels of phenolics and characterized F_9_ recombinant inbred lines (RILs) that accumulated contrasting levels of phenolics. In the Raskin lab, three lines accumulating high levels of anthocyanins were developed from existing red cultivars by visually selecting for deep-red color in tissue culture [15]. The lines were collectively named Rutgers Scarlet Lettuce (RSL). Selection from parent line cv. Firecracker was developed into RSL-NFR; from cv. Annapolis into RSL-NAR, and from cv. Grand Rapids Blackhawk into RSL-NBR [15]. The RSL lines accumulate the highest reported levels of phenolics in lettuce (>9% of dry weight), including chlorogenic acids, quercetin glycosides, and anthocyanins [15]. Consumption of RSL for 28 days in type 2 diabetic mouse models resulted in in vivo oral glucose tolerance improvement and decreased liver lipid levels compared to control [15,23]. Obese C57BL/6 mice fed a high-fat diet showed improvements in glucose tolerance after 13 weeks of supplementation with RSL powder, though other measured physiological parameters did not change significantly [24].

After generations of self-pollination, a spontaneous green mutant was observed among red seedlings of RSL-NAR [13,17]. This plant and its self-pollinated offspring, later named Green Super Lettuce (GSL) accumulated higher levels of quercetin glucosides than RSL-NAR, but almost no anthocyanins [13,17]. Later, a dark olive-green spontaneous mutant of GSL was isolated from self-pollinated GSL and named GSL Dark Green (GSL-DG) [17]. GSL-DG accumulated slightly higher levels of cyanidin than GSL. Homozygous GSL and GSL-DG plants harbor two different alleles of a CACTA transposon in the 5′ untranslated region of anthocyanidin synthase (*ANS*, also called leucoanthocyanidin dioxygenase, *LDOX*), a single-copy gene coding for an essential enzyme in anthocyanin biosynthesis [17].

**Figure 1:**
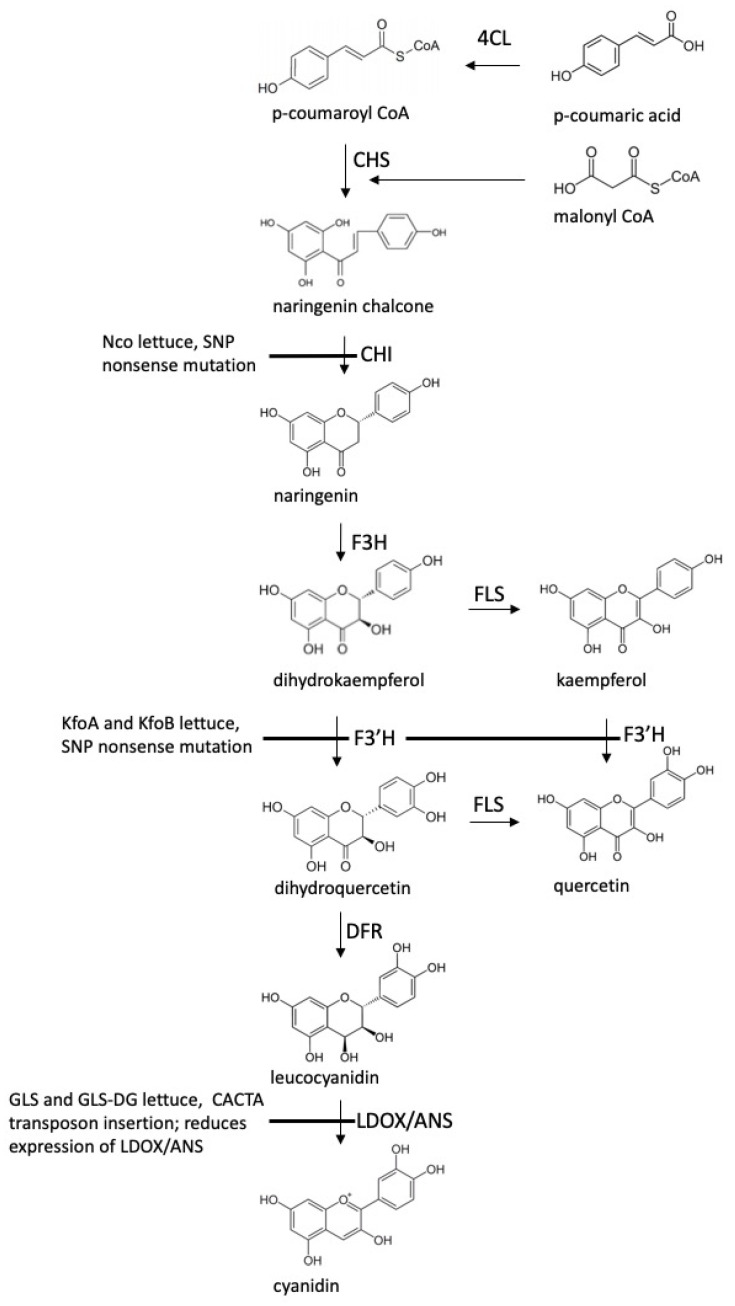
The flavonoid biosynthesis pathway showing enzymes altered in mutant lettuce lines GLS, GLS-DG, KfoA, KfoB, and Nco. Adapted from Saito et al. 2013 [35] and Gurdon et al. 2019 [16], and with additional information from Gurdon et al. 2019 [16] and Gurdon et al. 2021 [17]. Abbreviations: 4CL, 4-coumaric acid; CHS, chalcone synthase; CHI, chalcone isomerase; F3H, flavanone 3-hydroxylase; F3′H, flavonoid 3′-hydroxylase; FLS, flavonol synthase; OMT1, O-methyltransferase 1; DFR, dihydroflavonol 4-reductase; ANS, anthocyanidin synthase; LDOX, leucoanthocyanidin dehydrogenase.

In addition to the tissue culture-derived RSL-NFR line, three more lines with unusual flavonoid accumulation were developed from cv. Firecracker [16]. These lines were visually selected from an ethyl methanesulfonate (EMS)-mutagenized population, and then characterized genetically and phytochemically [16]. Two of the lines, kaempferol overproducer A (KfoA) and kaempferol overproducer B (KfoB), accumulate glycosides of kaempferol instead of quercetin and cyanidin due to independent mutations in the flavonoid-3′ hydroxylase (*F3′H*) gene, the product of which converts dihydrokaempferol into dihydroquercetin, and kaempferol into quercetin [16]. Kaempferol is a flavonol that has been shown to have anti–diabetic, pancreatic β-cell protecting, and anti-inflammatory effects in vitro and in vivo [25]. Both Kfo lines accumulate kaempferol at levels higher than other vegetables and fruits, apart from capers [16,26,27,28,29,30]. The third line, naringenin chalcone overproducer (Nco) accumulates glycosides of naringenin chalcone, a compound not previously reported in lettuce, and which has been shown to have anti-inflammatory, anti-allergic, and anti-obesity effects [16,31,32,33]. Nco carries a mutation in the chalcone isomerase (*CHI*) gene, the product of which converts naringenin chalcone to naringenin, which in wild-type lettuce gets further converted to flavonoids and anthocyanidins [16]. The major plant source of naringenin chalcone is tomato peel [34], where it accumulates to a similar level as in Nco lettuce. Figure 1 shows enzymes of the flavonoid biosynthesis pathway and alterations in GLS, GLS-DG, KfoA, KfoB, and Nco mutant lines.

While RSL [15], KfoA, KfoB, Nco [16], GSL, and GSL-DG [17] have all been thoroughly characterized phytochemically, plants subjected to these analyses were grown in plastic pots in growth chambers under cool fluorescent lights. In addition, plants were observed but not systematically characterized when grown in greenhouses with white supplemental lighting. It is well known that light quality has major effects on yield as well as flavonoid accumulation [36,37,38,39,40]. In lettuce [41] and wild-type *A. thaliana*, exposure to UV or blue light causes a reduction in biomass production but increases in accumulation of flavonoids. *A. thaliana* flavonoid biosynthesis mutants display more severe reactions to high UV light stress compared to the wild type. The *chalcone synthase* (*chs*), *chi* and *f3h* mutants (which entirely lack flavonols) had a higher sensitivity to UV light than *f3′h* mutants, which accumulate the flavonol kaempferol instead of quercetin present in the wild type plants [42]. As flavonoids absorb UV light and scavenge reactive oxygen species, mutants lacking flavonoids display increased photoinhibition and lipid and protein peroxidation as a result [43,44]. Greenhouse (natural light supplemented with high-pressure sodium lights) grown Nco, KfoA and KfoB lettuces produced comparable biomass to red cv. Firecracker, but produced less biomass under cool fluorescent lights, with Kfo (*f3′h*) performing better than Nco (*chi*) [16], similarly to *A. thaliana* [41].

Because environmental conditions impact plant growth and development, substantial variations have been observed in lettuce yield and quality when plants of the same cultivar were grown in different growing seasons [45,46]. To determine the performance of the eight newly developed, high-flavonoid lines in commercial field, we grew them over four growing seasons in the major lettuce production area located in the Salinas Valley of California. The high-flavonoid lines were compared in yield, resistance to diseases, postharvest quality, elemental composition, and content of pigments and common flavonoids to available dark-red parent lines cv. Annapolis (precursor of RSL-NAR, GSL, and GSL-DG) and cv. Firecracker (precursor of RSL-NFR, Nco, KfoA, and KfoB), as well as two unrelated red and dark red commercial cultivars (cv. Eruption, red Latin type, and cv. Merlot, dark-red leaf lettuce), two green commercial cultivars (cv. Darkland, dark green romaine type, and cv. Grand Rapids, light green leaf lettuce), and a light-green advanced breeding line (SM13-L2 [9]), leaf type) (Table 1, Figure S1).

## 2. Results

### 2.1. Biomass Production

Aboveground weight of plants at market maturity was measured in 2019 and 2021. In 2019, all Nco plants died during the growing season, and thus biomass production was not evaluated for this line. In 2021, only seven small Nco plants survived until harvest. Combined data from two years confirmed the highest biomass production in cv. Darkland, followed by the breeding line SM13-L2 (Table 2). The weight of all mutant lines, except Nco, was not significantly different from the weight of the remaining five control cultivars. The average weight of seven flavonoid mutant lines ranged from 353 g (RSL-NAR) to 571 g (GSL), while the weight of five control cultivars ranged from 389 g (cv. Firecracker) to 495 g (cv. Eruption). The average fresh weight of the surviving Nco plants (10 g) was substantially and significantly smaller than the fresh weight of plants from any other tested accession. The average height of plants ranged from 10 cm for Nco to 55 cm for RSL-NBR, the accession that started to bolt earliest from all tested accessions (Table 2). The average maximum width of plants ranged from 6 cm for Nco to 36 cm for SM13-L2 (Table 2). The effect size of the accessions for all three traits was significant, ranging from ω^2^ of 0.47 for plant weight to ω^2^ of 0.84 for plant height (Table S1).

### 2.2. Content of Pigments in Leaves

Overall chlorophyll content (SPAD-sqrt) and anthocyanin content (ACI-lb) were measured in 2019 and 2021. The overall chlorophyll content from two experiments was highest in the three accessions that visually appeared the darkest green, cv. Darkland (7.2), followed by GSL (7.0), and GSL-DG (6.7) (Table 2). The overall lowest chlorophyll content was observed in three light-green or yellow-green accessions: SM13-L2 (5.0), Nco (5.2), and cv. Grand Rapids (5.9).

**Figure 2:**
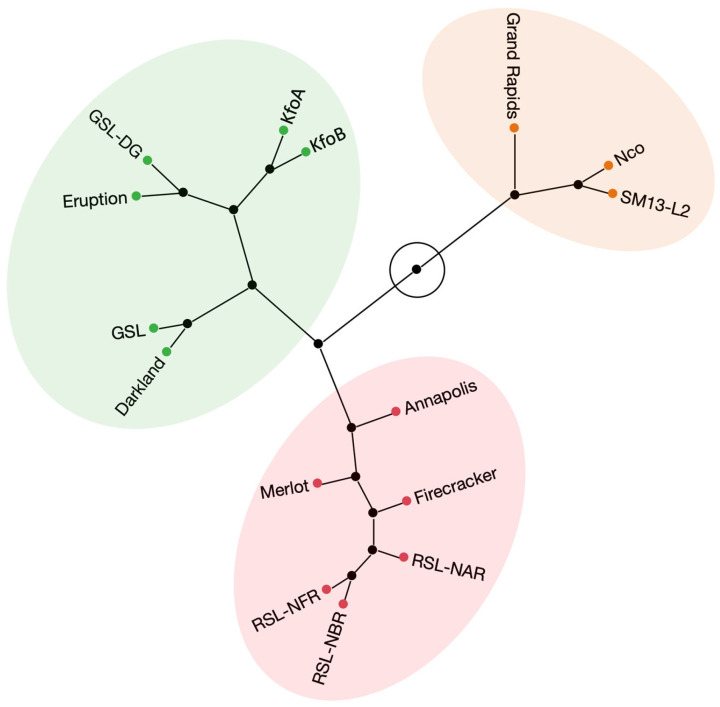
Hierarchical clustering analysis performed on the content of two pigments (chlorophylls and anthocyanins) evaluated with handheld meters on field-grown plants. Clustering was carried out using standardized data from two field experiments (Table 3) and the complete-linkage agglomerative method.

Over the two experimental years, the highest anthocyanin content was found in dark-red mutant lines RSL-NAR (8.0), RSL-NFR (7.8), and RSL-NBR (7.5), and dark-red cultivars Annapolis (7.9), Merlot (7.7), and Firecracker (7.1) (Table 2). Lower anthocyanin content was detected in red cv. Eruption (5.7). ACI-lb in green (GSL, GLS-DG, and cv. Darkland), and red-tinged (KfoA and KfoB) accessions ranged between 3.3 and 4.7. Light-green accessions (cv. Grand Rapids and SM13-L2) contained 1.8 to 1.9 ACI-lb, while the green-yellow mutant line Nco had the lowest anthocyanin content (1.3). Note that this anthocyanin quantification method provides a residual readout even in lines lacking cyanidins such as Nco. Accessions explained (ω^2^) approximately 0.37 and 0.92 of the variances in SPAD-sqrt and ACI-lb values, respectively (Table S1). Hierarchical clustering based on the values of anthocyanins and chlorophyll obtained by handheld meters grouped 15 accessions into three main clusters that approximately corresponded to visual color perception: a cluster containing six dark-red accessions, a second cluster containing three light-green accessions, and a third cluster consisting of green/dark green and red accessions (Figure 2). Though clustering of red cv. Eruption with green and dark-green accessions may seem unexpected, it is not surprising, as this cultivar contained substantially lower levels of anthocyanins (ACI-lb of 5.7) than accessions in the dark-red cluster (ACI-lb from 7.1–7.9) (Table 2).

### 2.3. Resistance to Diseases

Field-grown plants were assessed at market maturity for their resistance to downy mildew (DM, *Bremia lactucae*) in 2019 and 2020, to Impatiens necrotic spot virus (INSV) in 2020, 2021 and 2022, and to lettuce drop (LD, *Sclerotinia minor*) in 2020 and 2021. In the two years when resistance to DM was evaluated, the natural DM infection was very low. The average rating scores ranged from 0.03 (cv. Grand Rapids and KfoA) to 1.18 (RSL-NFR) on the 0–5 evaluation scale (Table 2). Due to this very low disease pressure, differences among accessions were not significant (LogWorth = 0.4 and ω^2^ = 0.02 for the effect size of accessions, Table S1).

The average incidence of naturally occurring INSV evaluated over three years ranged from 3% (Nco and RSL-NBR) to 24% (KfoA) (Table 2). The accessions’ effect size was ω^2^ = 0.08 (LogWorth = 1.5) (Table S1), with significant difference observed only between cv. Eruption (4% incidence) and KfoA. The differences in resistance between Nco and KfoA and between RSL-NBR and KfoA were not significant due to a higher variance in INSV incidence detected on Nco and RSL-NBR lines than on cv. Eruption.

Two experiments were performed in the field artificially infected with sclerotia of *S. minor* to assess the accessions’ resistance to LD. The average disease incidence ranged from 19% (cv. Eruption) to 72% (cv. Grand Rapids and KfoB) (Table 2). The accessions’ effect size for this disease was larger than for other two evaluated diseases, reaching a value of ω^2^ = 0.30 (LogWorth = 6.0) (Table S1).

### 2.4. Postharvest Quality

Two major aspects of lettuce postharvest quality were evaluated on plants harvested in 2019: tissue deterioration in modified atmosphere packaging (MAP) and enzymatic discoloration assessed on leaf disks. The area under the deterioration progress stairs (AUDPS) scores for tissue deterioration ranged from 17 (least deterioration, cv. Darkland and SM13-L2) to 35 (KfoA) (Table 2). Statistically significant differences (at *p* = 0.05) were observed only between two accessions with the lowest rate of deterioration (cv. Darkland and SM13-L2) and those with the most rapid deterioration (cv. Annapolis, KfoA, and KfoB).

When enzymatic discoloration was evaluated on leaf disks, the least discoloration was observed on breeding line SM13-L2 (area of 7 mm^2^), while the most profound was observed on cv. Merlot (92 mm^2^), KfoB (93 mm^2^), KfoA (94 mm^2^), and cv. Eruption (95 mm^2^) (Table 2). RSL-NAR was not evaluated for enzymatic discoloration, while Nco was not evaluated for any postharvest quality, as none of the plants survived in the field in 2019. The total variance in trait values accounted for by the effect of accessions was higher for enzymatic discoloration (ω^2^ = 0.71, logworth = 11.7) than for tissue deterioration (ω^2^ = 0.44, logworth = 2.4) (Table S1).

### 2.5. Elemental Composition

Elemental composition in dried pooled leaf samples was determined in 2022. Out of the 31 elements analyzed in dried tissue samples (Table 3), eight elements did not reach detection threshold levels and are not reported here. From the remaining 23 elements, the largest effect size was detected for Al (ω^2^ = 0.91, logworth = 13.5) and Mo (ω^2^ = 0.91, logworth = 13.4) (Table S1). Contrastingly, the smallest, though still significant, effect size was detected for the content B and Zn (both ω^2^ = 0.28, logworth = 1.5). When the content of elements in all accessions was compared, the accession with the highest content of most elements was Nco (maximum content was recorded for 13 elements), followed by cv. Eruption and RLS-NBR (both have the maximum content of three elements). When compared to the overall mean of all accessions, the most highly elevated contents of elements in Nco were Ti (489% of the overall mean), Mo (469%), Al (444%), Fe (428%), Li (386%), Cr (339%), Ba (266%), and Si (251%). Conversely, cv. Darkland had the most frequently occurring minimum content of elements (minimum content recorded for 11 elements), followed by breeding line SM13-L2 (minimum content of seven elements), and GLS (minimum content of two elements)

**Figure 3:**
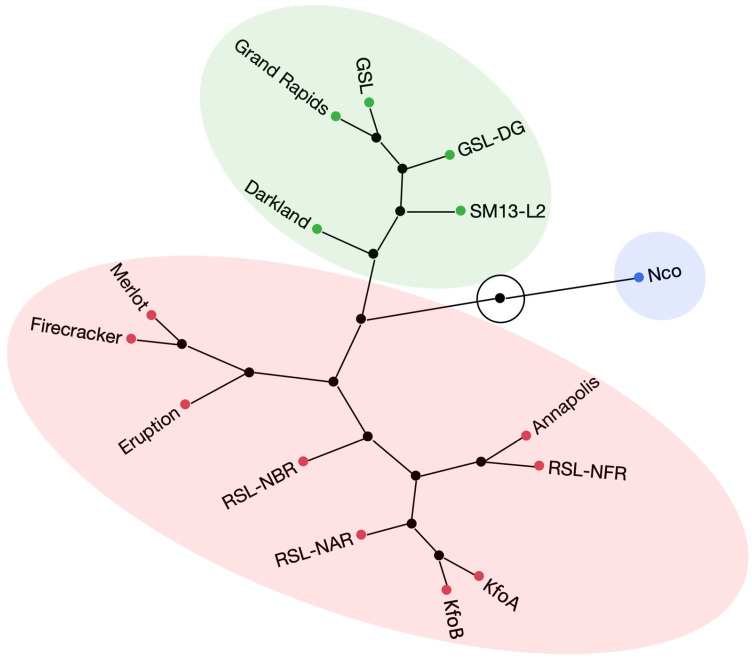
Hierarchical clustering analysis performed on the mineral element composition in dried leaf tissue. Clustering was carried out using standardized data from 23 mineral elements (Table 3) and the complete-linkage agglomerative method.

Hierarchical clustering analysis based on the content of 23 elements revealed three major clusters. As expected from the content of individual elements, Nco clustered separately from all other accessions (Figure 3). The second cluster contained five green accessions, while the third cluster consisted of six dark red accessions, one red accession, and two kaempferol accumulating mutant lines (KfoA and KfoB). It is interesting to note that except for naringenin (Nco)- and kaempferol (KfoA and KfoB)-accumulating mutant lines, clustering of all other accessions matched closely with their visual color perception, even though the hierarchical clustering analysis was based on mineral composition.

### 2.6. Flavonoid-Overproducing Lines Accumulate Higher Amounts of Flavonoids Compared to Parental Lines and Controls

Lettuce leaf samples harvested from the field were lyophilized, ground, and subjected to an acid hydrolysis extraction procedure that converted glycosylated flavonoids into aglycones. Significant differences were observed between the three growing years tested—2019, 2020, and 2021.

**Figure 4:**
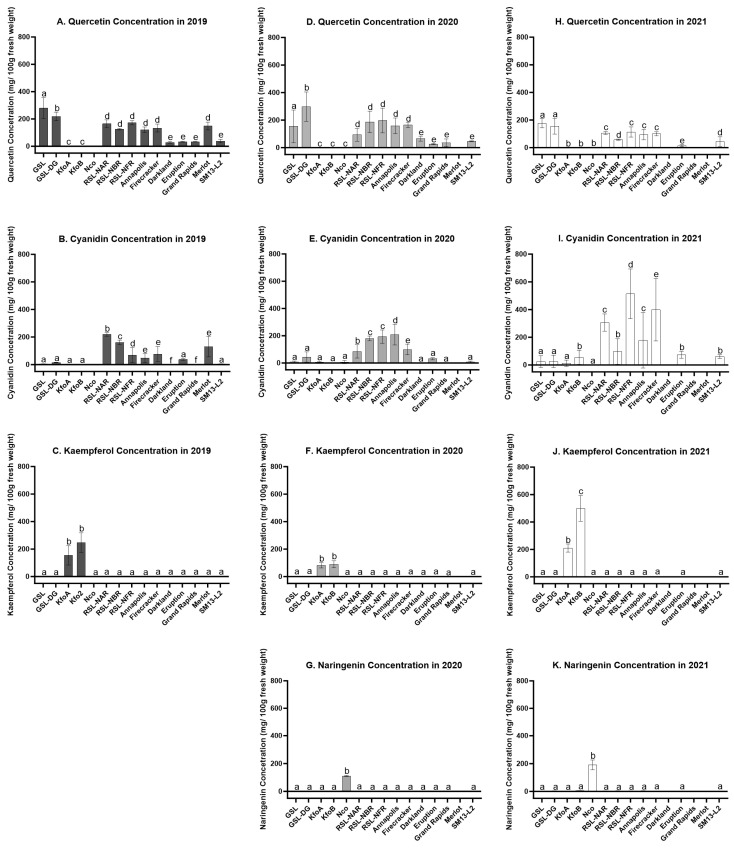
Phytochemical accumulation of four flavonoids from 15 lettuce accessions detected using UPLC-MS/MS. n = 4 for 2019, n = 3 for 2020 and 2021, significance is calculated using *p*-value 0.05, each bar is labeled with a letter indicating significance. Cultivars that were not grown in a specific year do not have a significance letter assigned. (**A**–**C**) Average content of quercetin, cyanidin, and kaempferol in mg/100 g fresh weight in 2019, (**D**–**G**) Average content of quercetin, cyanidin, kaempferol, and naringenin (converted from naringenin chalcone during extraction) in mg/100 g fresh weight in 2020. (**H**–**K**) Average content of quercetin, cyanidin, kaempferol, and naringenin in mg/100 g fresh weight in 2021.

GSL and GSL-DG lines, containing mutations in *ANS*, accumulated significantly higher levels of quercetin than all other tested accessions including RSL-NAR, from which both GSL and GSL-DG were derived. Based on data from the three field experiments, GSL accumulated on average 204 mg/100 g FW of quercetin, GSL-DG accumulated 224 mg, while RSL-NAR (parental line of GLS) accumulated 129 mg. Other dark-red cultivars and mutant lines accumulated on average 124 mg (RSL-NBR) to 161 mg (RSL-NFR) of quercetin. Quercetin content was even lower in red (cv. Eruption, 25 mg), dark-green (cv. Darkland, 47 mg), and light-green (cv. Grand Rapids and SM13-L2, 34 mg and 43 mg, respectively) accessions. KfoA, KfoB, and Nco mutant lines accumulated only negligible amounts of quercetin (<1 mg). (Figure 4).

In line with previous data from controlled environment conditions [13,15,16,17], the three RSL lines accumulated either statistically higher or similar concentrations of cyanidin than their parental cultivars (RSL-NAR 203 mg/100 g FW vs. cv. Annapolis 145 mg, RSL-NFR 259 mg vs. cv. Firecracker 196 mg, and RSL-NBR 146 mg—the parental line was not available for testing). All other control accessions and mutant lines accumulated significantly less cyanidin (<50 mg averaged over the three growing seasons). Kaempferol mutants KfoA and KfoB, with point mutations in F3′H were the only lines that accumulated kaempferol, which ranged from 83 mg/100 g FW to 210 mg in KfoA and 90 mg to 499 mg in KfoB. Similarly, naringenin, which is converted from naringenin chalcone during extraction, was detected only in Nco plants (111 mg/100 g FW to 191 mg). Naringenin concentration was either undetectable or significantly lower (<5 mg) in the other 14 tested accessions.

**Figure 5:**
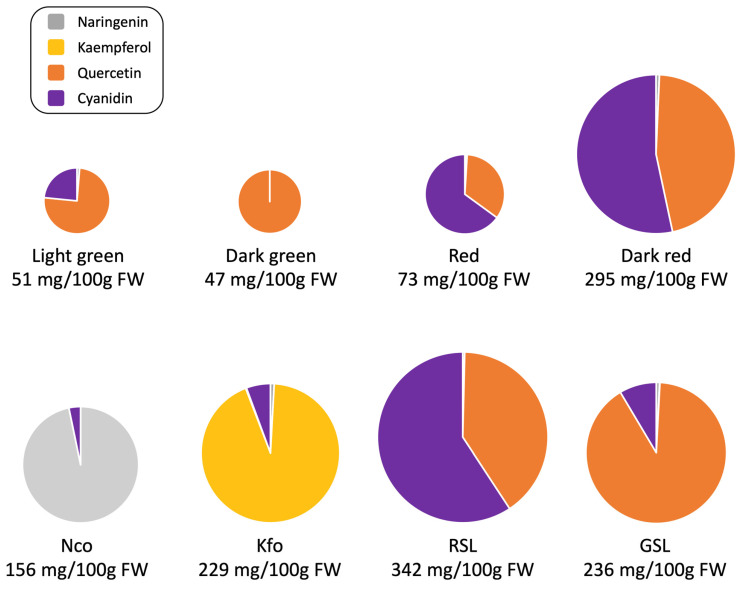
The flavonoid profiles of the newly developed, high-flavonoid lines and control accessions grown in the field. Average values of flavonoids content from three years (Figure 4) were used to draw respective profiles. The total content of analyzed flavonoids is shown for each group. Grouping of control accessions into four color groups was based on their visual perception (Table 1). Light-green group (cv. Grand Rapids and SM13-L2), dark-green group (cv. Darkland), red group (cv. Eruption), dark-red group (cvs. Annapolis, Firecracker, and Merlot).

The flavonoid profiles of the eight newly developed high-flavonoid lines grown in the field (Figure 5) were similar to those of the same lines grown under controlled environment conditions. However, the absolute levels of measured flavonoids varied significantly between years (Figure S2). For example, RSL-NFR accumulated 7.3-fold more cyanidin in 2021 (514 mg/100 g FW) than in 2019 (70 mg). Similarly, high variation was seen for the kaempferol levels in KfoB, which ranged from 90 mg in 2020 to 499 mg in 2021. Less variation was observed in the accumulation of quercetin in GSL and GSL-DG and naringenin in Nco lines. Despite year-to-year variation in the accumulation of flavonoids, the effect size of accessions was substantially larger than the effect size of experiments (Table S1). Values of ω^2^ for accessions ranged from 0.41 (logworth = 10.7) for cyanidin to 0.88 (logworth = 29.1) for naringenin. The ω^2^ values for the effect size of experiments were in the range from 0.01 (logworth = 2.1) for naringenin to 0.09 (logworth = 5.0) for cyanidin.

**Figure 6:**
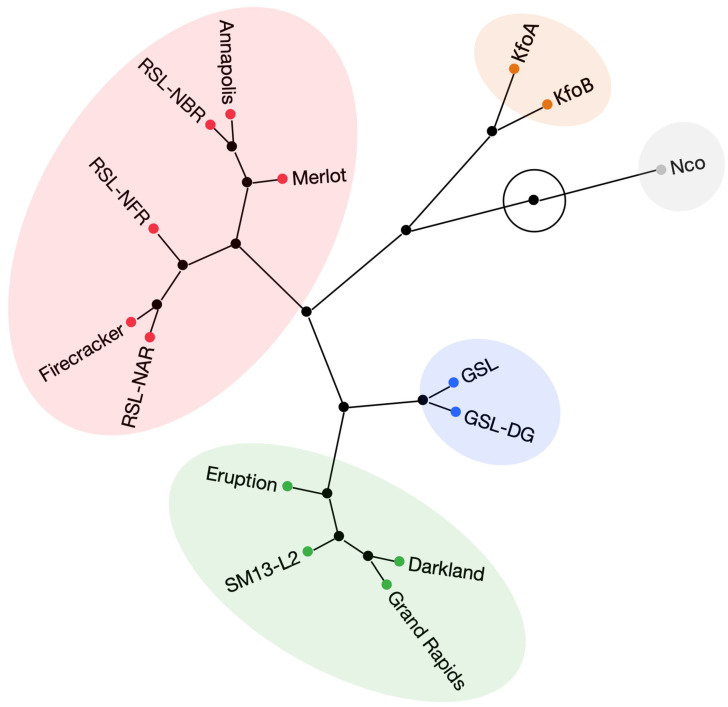
Hierarchical clustering analysis performed on the content of four flavonoids determined in harvested leaf tissue. Clustering was carried out using standardized data of the average content of naringenin, kaempferol, quercetin, and cyanidin (Figure 4) and the complete-linkage agglomerative method.

Hierarchical clustering based on the content of four flavonoids identified five clusters (Figure 6). The largest cluster contained three dark red accessions (cvs. Annapolis, Firecracker, and Merlot) plus three dark-red mutant lines (RSL-NAR, RSL-NBR, and RSL-NFR). The second-largest cluster consisted of three green accessions (cvs. Darkland and Grand Rapids, breeding line SM13-L2) and red cv. Eruption. The remaining three clusters contained a group of two mutant lines accumulating elevated levels of quercetin (GSL and GSL-DG), kaempferol (KfoA and KfoB), and naringenin (Nco).

## 3. Discussion

Prior biochemical and genetic characterizations of RSL and other high-flavonoid red and green lettuce lines were performed under controlled greenhouse and growth chamber conditions [15,16,17,36,37]. This report examines the biochemical and agronomic characteristics of these lines grown under standard field conditions in the areas of California suitable for commercial lettuce production. We grew eight flavonoid-overproducing lines—three cyanidin-rich (RSL-NAR, RSL-NBR, and RSL-NFR), two quercetin-rich (GSL and GSL-DG), two kaempferol-rich (KfoA and KfoB), and one naringenin chalcone-rich (Nco)—under field conditions and compared them with two parental lines and five additional accessions of varying color. Cv. Darkland and SM13-L2 had the overall highest biomass production, but all other control accessions were similar in their biomass production to the mutant lines (except for Nco). In general, our data confirmed that RSL, GSL, GSL-DG, Kfo, and Nco lines accumulated similar or higher levels of flavonoids in the field as observed previously under controlled environment conditions.

RSL lines are cell culture-selected, dark-red lettuces that overproduce anthocyanins, as well as quercetin and other intermediates of the flavonoid pathway [15]. They have not been genetically characterized. Field-grown RSL lines generally produced higher levels of polyphenols than their parental lines cv. Annapolis (NAR) and cv. Firecracker (NFR). Among three RSL lines, RSL-NFR accumulated on average the most anthocyanin, specifically cyanidin. Similarly, *ans* mutants GSL and GSL-DG accumulated significantly more quercetin than their precursor (cv. Annapolis) or other tested lines. Kfo and Nco lines (*f3′h* and *chi* mutants, respectively) accumulated solely kaempferol and naringenin chalcone, while levels of other flavonoids were not detectable when analyzed using UPLC-MS/MS. Kaempferol accumulation in two Kfo lines ranged from 83 mg/100 g FW to 499 mg/100 g FW, which is substantially more than was reported in other high-kaempferol-producing vegetables, such 11.8 mg in leeks (*Allium porrum*), 47.0 mg in kale (*Brassica oleracea* var. *acephala*), 35.1 mg in watercress (*Nasturtium officinale*), and up to 60.4 mg/100 g FW in rocket (*Eruca sativa*) [27,30,47]. Similarly, Nco accumulated naringenin chalcone at levels much higher than any other reported fruits or vegetables, from 111 mg/100 g FW to 191 mg/100 g FW. Naringenin chalcone has primarily been described from tomato fruit peel, e.g., cherry tomato (*Solanum lycopersicum* var. *cerasiforme*) contains up to 4.0 mg/100 g FW of naringenin [48]. Though the content of flavonoids in the mutant lines grown in the field was high, we observed a substantial difference in their content across years. For example, RSL-NFR cyanidin accumulation was 70 mg/100 g FW in 2019 but increased 7.3-fold to 514 mg/100 g FW in 2021. These differences could be a result of variations in temperature, light, or other environmental conditions and stresses occurring during field cultivation [49].

When the eight mutant lines grown in the field were compared to the seven control accessions (two of them parental lines), biomass production (fresh weight) and plant size were acceptable for all mutant lines, except for Nco, which produced only a limited number of very small plants (Table 2). There was no indication of pests or pathogens causing poor germination or slow growth of this line. When Nco and cv. Firecracker germination was tested in petri dishes using wet filter paper, seeds of Nco germinated about two days later and had a lower germination rate (80% vs. 100%). This difference alone, however, does not explain why only a few small plants were produced under field conditions. The *chi* mutation leading to naringenin chalcone accumulation (Figure 1) causes a complete lack of flavonoids, compounds protecting plants from damaging radiation. The lack of these compounds results in higher sensitivity to UV irradiation compared to wild-type plants in *A. thaliana*, as well as reduced growth [42,43,44]. When cultivated under controlled environment conditions using fluorescent lights (UV light intensity 0.4 mol m^−2^ d^−1^), Nco plants grew slower than those of cv. Firecracker [16]. In our field experiments, Nco plants accumulated a substantially higher content of certain elements than other tested accessions, such as Ti (489% of the overall mean), Mo (469%), Al (444%), Fe (428%), Li (386%), Cr (339%), Ba (266%), and Si (251%) (Table 3). Though it cannot be determined whether this elevated content of elements was related to a small plant size, it is known that an excess of certain elements may have a negative effect on plant germination, growth, and/or development. For example, high content of iron, a microelement necessary for plant photosynthesis, can reduce germination, cause severe morphological and physiological disorders, retard carbon metabolism, and lead to ferroptosis [50]. From the other seven mutant lines, RSL-NBR may be transitioning from vegetative to the reproductive stage earlier than other tested accessions, as was indicated by its greater height (Table 2). Because premature bolting is an undesirable trait, this line should be retested in commercial production conditions for the earliness of bolting [51,52].

In general, elevated flavonoid levels have been linked to increased resistance against bacterial, fungal and viral pathogens in multiple crops, though the mechanism of action and contribution of individual metabolites to resistance is frequently unclear [53,54]. Reaction of the mutant lines to the three pathogens causing DM, LD, and INSV was generally in the acceptable range. Though low DM pressure did not allow the detection of statistically significant differences in the accessions’ reaction to the pathogen, the order of average DM scores (Table 2) agreed with previous observations. Cvs. Grand Rapids (rating = 0.03) and Merlot (rating = 0.10) were among the most resistant to the disease when more than 800 accessions were evaluated for polygenic resistance in multiple field trials [55,56]. Similarly, the SM13-L2 line (rating = 0.10) was developed to have a high polygenic resistance to DM [57]. Conversely, cv. Darkland (rating = 0.79), when tested in more than 40 field trials, ranked approximately in the midrange of evaluated accessions [55,56]. Considering the aforesaid good match between the current and previous results, mutant lines KfoA (rating = 0.03) and RSL-NAR (rating = 0.04) may have a high level of field resistance to DM. However, if field production of the flavonoid mutant lines is considered, GLS-DG (rating = 1.01) and RSL-NFR (rating = 1.18) may need to be retested for their resistance to DM at a higher disease pressure.

Reaction to *S. minor*, the fungus causing LD, was evaluated in two experiments in an artificially infected field (Table 2). Though no lettuce accession is known to be immune to LD [58], cv. Eruption was the most resistant from the diversity panel of ~500 accessions tested in multiple field trials [59]. This cultivar carries several resistance loci [60] against LD. None of the flavonoid mutant lines had disease incidence as low as cv. Eruption (19%), while the next-lowest incidence was observed for Nco (47%) and RSL-NAR (52%). The highest LD incidence among mutant lines was recorded for KfoA (71%) and KfoB (72%), values similar to their parental line cv. Firecracker (68%). Genome-wide association mapping results revealed a frequent, nonrandom colocation of loci for anthocyanin content and resistance to LD [59], with *ANS* being suggested as one of the candidate genes for functional studies to ascertain the involvement of anthocyanins in lettuce resistance to LD. However, cv. Eruption had significantly lower total anthocyanin content (ACI-lb) and lower cyanidin (and quercetin) content than the RSL lines and cv. Annapolis in all tested years (Table 1, Figure 2); thus, genetic loci other than *ANS* play a substantial role in LD resistance.

INSV is currently one of the most devastating diseases on lettuce occurring in the major lettuce producing area of the US, located in the Salinas Valley of California [61]. Despite extensive testing, no lettuce accession immune to the disease has been detected [62]. However, cv. Eruption has shown one of the highest levels of a partial resistance to INSV from over 500 lettuce accessions tested in 18 field experiments over eight years [61,63]. The current study confirmed low disease incidence in cv. Eruption (4%) and in cv. Annapolis (5%) (Table 2). Even lower INSV incidence was recorded in Nco and RSL-NBR (both 3%). Resistance data obtained on Nco need to be considered as preliminary because of the small number of evaluated plants and also due to delayed development of plants that may have affected disease incidence. INSV incidence in other mutant lines was similar to those in control accessions. The multiple linear regression model that used INSV incidence as a dependent variable and the content of anthocyanins, chlorophyll, plant development, and thrips (INSV vector) feeding damage as independent variables revealed a significant negative correlation between the content of anthocyanins and disease incidence [63]. It was not determined, however, if anthocyanins play a direct role in resistance to INSV, or if both resistance and pigment content are related to yet another trait that may affect thrips feeding preference.

Lettuce is a highly perishable product; therefore, postharvest quality is one of the major considerations when evaluating new breeding lines [10]. Among the tested mutant lines, the highest tissue deterioration on freshly cut lettuce stored in MAP was observed on KfoA (AUDPS of 35), KfoB (AUDPS of 32), and RSL-NFR (AUDPS of 32) (Table 2). These values are similar to those for cv. Annapolis (AUDPS of 34), whose rate of postharvest deterioration is still acceptable to the lettuce processing industry.

Extensive enzymatic discoloration (pinking and browning) occurring on stems and midribs makes the product less attractive to consumers [64]. An assay based on leaf disks has been proposed to identify genotypes with low discoloration potential [65]. A relatively high discoloration area was detected in KfoA (94 mm^2^) and KfoB (93 mm^2^), which is somewhat higher (though not significantly) than discoloration detected in their parental line cv. Firecracker (75 mm^2^) (Table 2). The lowest potential for enzymatic discoloration was detected in breeding line SM13-L2 (7 mm^2^) but also in RSL-NBR (19 mm^2^) and GSL (22 mm^2^).

Hierarchical clustering based on pigments (Figure 2), mineral element content (Figure 3), and concentration of flavonoids (Figure 6) revealed a substantial match with visual perception of lettuce color. Somewhat surprisingly, however, red cv. Eruption grouped with green accessions when the content of pigments (anthocyanins and chlorophyll) (Figure 2) or flavonoids (Figure 6) was used for clustering. This may have happened because leaves of cv. Eruption contain substantially less quercetin and cyanidin than was detected in dark-red accessions (Figure 4 and Figure 5).

## 4. Materials and Methods

### 4.1. Plant Material

Experiments were performed to determine whether the eight lines that produce high levels of flavonoids in controlled environment conditions or are mutants in flavonoid biosynthesis (collectively referred to as flavonoid mutants [15,16,17]) can maintain the elevated production of flavonoids under field conditions while yielding a similar biomass compared to the commercial lines. The flavonoid mutants RSL-NFR, RSL-NBR, KfoA, KfoB, and Nco phenotypically belong to the loose-leaf type of lettuce that does not form a compact “head” of tightly packed leaves at market maturity, such as is typical for iceberg, romaine, butterhead, Latin, and Batavia types [9]. The flavonoid mutants RSL-NAR, GSL, and GSL-DG are somewhat different, phenotypically resembling a transitional type between loose-leaf and romaine-like type. In addition to the tested lines, seven control accessions were grown in the same experiments: parental cultivars (Annapolis and Firecracker), released cultivars (Darkland, Eruption, Grand Rapids, and Merlot), and an advanced breeding line—SM13-L2 [57]. These control accessions were selected to include phenotypes of different color, ranging from light green, to dark green, red, and dark red (Table 1, Figure S1).

### 4.2. Field Cultivation

Four field experiments were performed in Salinas, California. The experiments were carried out in 2019, 2020, 2021, and 2022 using randomized complete block design (RCBD) with three replications (i.e., three replicated plots per accession). Plants were grown on ~1.0 m-wide and ~0.25 m-high raised beds with two parallel seedlines in the center of each bed at a distance of ~0.28 m from each other. Approximately three weeks after germination, plants were thinned to the final distance of about 0.30 m between adjacent plants within each seedline. After thinning, each plot contained approximately 25 plants of the same accession, which were then grown to market maturity. Experimental fields were maintained using standard agricultural practices for the area [9]. Because only a minimal natural precipitation occurs during lettuce growing season (Table S3), all fields were irrigated using an overhead sprinkler system. The fresh, aboveground weight of ten individual plants from the middle of each plot was evaluated at market maturity in 2019 and 2021. In 2021, five plants per accession and replication (15 plants per accession in total) were evaluated for height (from soil level to the plant top) and width (the widest diameter of the plant).

### 4.3. Disease Resistance Evaluation

Resistance to downy mildew (DM), lettuce drop (LD), and Impatiens necrotic spot virus (INSV), three of the most predominant and economically important diseases in the growing region, was evaluated in 2019 (DM), 2020 (DM, LD, INSV), 2021 (LD, INSV), and 2022 (INSV). Resistance to DM and INSV was based on a natural occurrence of the pathogens, while resistance to LD was evaluated in an artificially infected field.

#### 4.3.1. Resistance to Downy Mildew

Resistance to DM, the disease caused by oomycete *Bremia lactucae*, was evaluated at market maturity on all plants in a plot using the 0 (no disease) to 5 (severe plant infection) scale that takes into consideration both the disease incidence and severity [66].

#### 4.3.2. Resistance to Impatiens Necrotic Spot Virus

INSV is transmitted by the western flower thrips (*Frankliniella occidentalis* Pergande) [61]. Disease incidence evaluated at market maturity was calculated as a percentage of plants that were dead due to INSV infection or showed typical INSV symptoms [63] from the total number of plants in a plot.

#### 4.3.3. Resistance to Lettuce Drop

Lettuce drop disease occurring in the Salinas Valley is caused by the soil-dwelling fungus *Sclerotinia minor* [67]. To evaluate resistance to LD, plants were grown in an artificially infected field [58] using RCBD with three replications and agronomic practices typical for the growing area. Weekly evaluations of plots started at the first appearance of the disease and continued until market maturity [58]. The cumulative disease incidence for each plot was calculated as a percentage of dead or symptomatic plants from the total number of plants per plot prior to disease onset.

### 4.4. Quantification of Total Anthocyanins and Chlorophyll in the Field

Three representative, randomly selected plants per plot were evaluated for the content of chlorophyll and anthocyanins at market maturity in 2019 and 2022. The contents of chlorophyll (SPAD units) [68] and anthocyanins (ACI units) [69] were determined on the three largest, healthy leaves at the position of about 2 cm from a leaf tip using SPAD-502 (Konica Minolta Sensing, Tokyo, Japan) and ACM-200 plus (Opti-Sciences, Hudson, NH, USA) meters, respectively. Three measurements of SPAD and ACI taken on each of the three selected leaves were averaged. The average of these nine values (per plant) of chlorophyll and anthocyanins were transformed using square root (SPAD-sqrt) and binary logarithm (ACI-lb) functions, as previously recommended [70]. Transformed values from each of the three plants per plot were used for statistical analyses.

### 4.5. Evaluations of Postharvest Quality

Two aspects of postharvest quality were evaluated on plants harvested at market maturity in 2019: tissue deterioration and enzymatic discoloration [64]. Descriptions of postharvest quality evaluations are detailed in Section 4.5.1 and Section 4.5.2.

#### 4.5.1. Tissue Deterioration

To assess tissue deterioration, healthy leaves from ten plants per accession were cut into approximately 2.5 cm^2^ pieces, washed with a weak solution of sodium hypochlorite (0.0016 mol l–1) and then distilled water, dried with a food processing centrifuge, sealed into transparent plastic bags containing low O_2_ level (~1.5%) achieved by flushing bags with N_2_, and stored in dark at 5 °C [71]. Tissue in each plastic bag was visually evaluated for deterioration in weekly intervals, using the 0–10 scale that approximately expresses the percentage of deteriorated tissue divided by 10 and rounded to the nearest whole number [72]. After four weeks in cold storage, weekly evaluations for each bag were combined into a single score using the area under the deterioration progress stairs (AUDPS) approach [52]. This tissue processing, storing, and evaluation approach provides results that closely correlate with those obtained from modified atmosphere packaging (MAP) used by the US lettuce processing industry [71].

#### 4.5.2. Enzymatic Discoloration

Enzymatic discoloration of accessions was assessed using the modified leaf disk approach [65]. Healthy leaves of approximately the same age from middle of the harvested plants were used in this experiment. Six disks of 12 mm in diameter were cut from the top of the leaves with a cork borer, avoiding midribs and major leaf veins. Disks were put abaxial side down on a filter paper wet with deionized distilled H_2_O, placed into a sealed container to prevent evaporation, and kept in dark at 4 °C. After one week in cold storage, photographs of disks were taken and the size of the red/pink colored area around each leaf disk was measured using ImageJ2 v1.51 [73].

### 4.6. Determination of Leaf Elemental Composition

In 2022, leaves from three healthy plants per plot were collected at market maturity and combined into a single sample. Samples were oven-dried for 12 h at 70 °C before analyses of 31 chemical elements were performed by Wallace Laboratories (El Segundo, CA, USA). Standard analytical methods [74,75,76,77] were used to determine concentrations of aluminum (Al), arsenic (As), barium (Ba), boron (B), cadmium (Cd), calcium (Ca), chloride (Cl-), chromium (Cr), cobalt (Co), copper (Cu), iron (Fe), lead (Pb), lithium (Li), magnesium (Mg), manganese (Mn), mercury (Hg), molybdenum (Mo), nickel (Ni), nitrogen (N), phosphorus (P), potassium (K), selenium (Se), silicon (Si), silver (Ag), sodium (Na), strontium (Sr), sulfur (S), tin (Sn), titanium (Ti), vanadium (V) and zinc (Zn). All concentrations are reported in micrograms per gram of dry weight, except for N, reported as percentage of dry weight. Concentrations of eight elements (Ar, As, Co, Pb, Hg, Se, Sn, and V) that did not reach the detection threshold levels in a majority of accessions were not considered for further statistical analyses and are therefore not reported.

### 4.7. Flavonoid Quantification

Leaf tissue from plants grown in 2019, 2020, and 2021 field experiments was harvested at market maturity, weighted, lyophilized, and shipped to Rutgers University (New Brunswick, NJ, USA) for flavonoid quantification. Laboratory analyses were performed using analytical standards >98% purity quercetin 3-glucoside (16654), cyanidin chloride (94099), and ≥97% purity kaempferol (60010) purchased from Sigma (St. Louis, MO, USA), PTFE filters (0.45 μm, 89041-306) and tert-butylhydroquinone purchased from VWR (Radnor, PA, USA), and organic solvents purchased from Sigma Aldrich (St. Louis, MO, USA).

#### 4.7.1. Extraction and Acid Hydrolysis of Flavonoid Aglycones

Flavonoid aglycones were quantified using lyophilized leaves from the 15 tested lettuce cultivars over the course of three years (2019–2021). The leaves were stored at −80 °C, lyophilized and ground to a powder. An acid hydrolysis procedure based on Hertog et al. (1992) [26] and as described in Gurdon et al. (2019) [16] was performed to convert glycosylated flavonoids into respective flavonoid aglycones. In summary, 50 mg of leaf powder was added to a plastic tube, to which 4 mL of solvent (62.5 methanol:32.5 water and 2 g/L tert-butylhydroquinone) was added. The mixture was acidified with 1 mL of HCl, vortexed for several seconds, and kept at 90 °C for 2h. The volume of the extract was made up to 10 mL using 100% methanol and tubes were sonicated for 5 min. After centrifugation for 8 min at 2500 rpm, the extract was filtered through 0.45 μm PTFE filters for UPLC-MS/MS analysis.

#### 4.7.2. UPLC-MS/MS Analysis

Samples were separated and analyzed by a UPLC/MS system including the Dionex^®^ UltiMate 3000 RSLC ultrahigh-pressure liquid chromatography system, with a workstation with Thermo Fisher Scientific’s Xcalibur v. 4.0 software package (Waltham, MA, USA). After the photodiode array detector, the eluent flow was guided to a Q Exactive Plus Orbitrap high-resolution high-mass-accuracy mass spectrometer (MS). Mass detection was a full MS scan with low-energy collision-induced dissociation (CID) from 100 to 1000 *m*/*z* in positive-ionization mode with an electrospray (ESI) interface. The mass resolution was 140,000. Substances were separated on a PhenomenexTM Luna C8 reverse phase column.

#### 4.7.3. Flavonoid Aglycone Quantification

Putative formulas of polyphenols and other compounds were determined by performing isotope abundance analysis on the high-resolution mass spectral data with Xcalibur v. 4.0 software and reporting the best-fitting empirical formula. Quantification was based on external standards made with kaempferol, cyanidin chloride, and quercetin. Naringenin and luteolin were quantified using the kaempferol standard curve. Naringenin was quantified, as naringenin chalcone is converted to naringenin during acid hydrolysis (for evidence of Nco accumulating naringenin chalcone, see [16]). Database searches were performed using reaxys.com (RELX Intellectual Properties SA, London, UK) and SciFinder (American Chemical Society, Washington, DC, USA).

Concentrations of all flavonoids are reported in milligrams per 100 g of fresh weight (FW) of tissue.

### 4.8. Statistical Analysis

Data were evaluated using analysis of variance (ANOVA) followed by Tukey’s HSD post hoc test for multiple comparisons. The effect size for main factors (accessions and experiments) was estimated from ANOVA by the means of omega squared (ω^2^) statistics. Hierarchical clustering was carried out using the average values of traits for each accession. All statistical analyses were performed with JMP Pro 17 (SAS Institute, Cary, NC, USA). Reported logworth values are -log10 transformations of *p*-values, so logworth of 2 is equivalent of *p*-value of 0.01, and logworth of approximately 1.301 is equivalent of *p*-value = 0.05.

## 5. Conclusions

The current study confirmed the stability of flavonoid production in mutant lines, with all lines producing as much or higher quantities of specific flavonoids as in controlled environment conditions. Seven out of the eight tested high-flavonoid lines (except Nco) produced the amount of biomass comparable to leaf-type cultivars used as controls. These lines may be considered for field production, particularly as a novelty item in baby-leaf or spring-mix products. The high-flavonoid lettuce lines may appeal to customers based on the perceived health benefits of higher levels of antioxidants compared to lettuces found typically on the market. The Nco line is not suitable for field production due to its slow growth and poor survival. It is not known if the line growth and survival under field conditions is related to the mutation in the flavonoid pathway or other mutation(s) in the genome caused by EMS. The high-flavonoid mutants tested in this study may be used to introgress novel genes into the lettuce gene pool by crossing mutant lines with genotypes with high yield, resistance to biotic and abiotic factors, and superior postharvest quality. Such an approach may allow the development of romaine cultivars producing high levels of kaempferol, quercetin, or cyanidin.

## Figures and Tables

**Table 1:** Description of eight flavonoid mutant lines and seven control accessions evaluated in field experiments.

Accession	Category	Origin	Color	Horticultural Type
RSL-NAR	Mutant line	Tissue culture derivative of Annapolis	Dark red	Romaine like
RSL-NBR	Mutant line	Tissue culture derivative of Grand Rapids Blackhawk	Dark red	Loose-leaf
RSL-NFR	Mutant line	Tissue culture derivative of Firecracker	Dark red	Loose-leaf
GSL	Mutant line	Natural mutant of RSL-NAR	Green with red patches	Romaine like
GSL-DG	Mutant line	Natural mutant of GSL	Dark green	Romaine like
KfoA	Mutant line	EMS induced mutant of Firecracker	Green with red tingle	Loose-leaf
KfoB	Mutant line	EMS induced mutant of Firecracker	Green with red tingle	Loose-leaf
Nco	Mutant line	EMS induced mutant of Firecracker	Yellow-green	Loose-leaf
Annapolis	Parental line	Released cultivar	Dark red	Romaine
Firecracker	Parental line	Released cultivar	Dark red	Loose-leaf
Darkland	Control	Released cultivar	Dark green	Romaine
Eruption	Control	Released cultivar	Red (mostly)	Latin
Grand Rapids	Control	Released cultivar	Light green	Loose-leaf
Merlot	Control	Released cultivar	Dark red	Loose-leaf
SM13-L2	Control	Grand Rapids × Iceberg	Light green	Loose-leaf

**Table 2:** Field biomass production, pigments content, resistance to diseases, and postharvest quality of flavonoid mutant lines and control accession.

Accession	Plant Weight (g)	Plant Height (cm)	Plant Width (cm)	Chlorophyll (SPAD-sqrt)	Anthocyanins (ACI-lb)	Downy Mildew (Rating)	Lettuce drop (Incidence %)	INSV (Incidence %)	Tissue Deterioration (AUDPS)	Enzymatic Discoloration (Area mm^2^)
RSL-NAR	353 c	29 bc	23 bc	6.5 a	8.0 a	0.04	52 a–c	10 ab	29 ab	-
RSL-NBR	433 c	55 a	25 b	6.4 a	7.5 a	0.36	55 a–c	3 ab	25 ab	19 de
RSL-NFR	434 c	31 b	24 bc	6.4 a	7.8 a	1.18	61 ab	11 ab	32 ab	61 a–c
GSL	571 bc	26 bc	23 bc	7.0 a	3.4 d–f	0.35	64 ab	13 ab	26 ab	22 c–e
GSL-DG	510 bc	29 bc	20 c	6.7 a	4.0 de	1.01	55 a–c	11 ab	30 ab	63 ab
KfoA	411 c	27 bc	27 b	6.1 ab	3.3 d–f	0.03	71 a	24 a	35 a	94 a
KfoB	500 c	25 cd	24 bc	6.1 ab	4.7 cd	0.73	72 a	10 ab	32 a	93 a
Nco	10 d	10 e	6 d	5.2 ab	1.3 e–g	-	47 a–c	3 ab	-	-
Annapolis	433 c	30 b	23 bc	6.7 a	7.9 a	0.64	33 bc	5 ab	34 a	67 ab
Darkland	965 a	29 bc	21 bc	7.2 a	3.4 d–f	0.79	65 ab	21 ab	17 b	49 b–d
Eruption	495 c	20 d	23 bc	6.4 a	5.7 bc	0.85	19 c	4 b	22 ab	95 a
Firecracker	389 c	31 b	23 bc	6.4 a	7.1 ab	0.40	68 ab	11 ab	25 ab	75 ab
Grand Rapids	447 bc	29 bc	31 ab	5.9 ab	1.9 fg	0.03	72 a–c	12 ab	25 ab	46 b–e
Merlot	435 c	30 bc	26 b	6.2 ab	7.7 a	0.10	62 a–c	15 ab	28 ab	92 a
SM13-L2	764 ab	28 bc	36 a	5.0 b	1.8 g	0.10	54 ab	8 ab	17 b	7 e

Mean values within a column followed by different letters are significantly different at *p* ≤ 0.05 using Tukey’s HSD test. A range of letters, e.g., “a–c” indicates the first and the last letter of the range in alphabetic order. Values for downy mildew rating were not significantly different (at *p* ≤ 0.05). Missing values are indicated by dashes. Mean values from individual years for the traits evaluated in more than one experiment are in Table S2.

**Table 3:** Mineral element composition of flavonoid mutant lines and control accessions grown under field conditions.

Accession	N	P	K	Ca	S	Mg	Fe	B	Cl-	Mn	Zn	Cu
RSL-NAR	3.40 b–d	6155 a–c	45,984 b–d	11,236 ab	2296 bc	4975 bc	225 b	30.3 a	30,629 a–d	157 a	31 ab	5.5 b
RSL-NBR	4.04 ab	3533 e	37,882 cd	11,158 ab	2505 ab	7680 a	300 b	26.6 ab	38,655 a	107 a–c	32 ab	7.2 b
RSL-NFR	4.13 ab	5658 a–d	49,574 a–c	8960 ab	2294 bc	4737 bc	210 b	28.9 ab	38,032 ab	107 a–c	32 ab	7.6 b
GSL	3.72 ab	5079 b–e	31,848 d	8547 ab	2246 bc	3595 bc	242 b	28.0 ab	14,483 g	103 a–c	31 ab	4.9 b
GSL-DG	3.62 a–c	5389 a–e	35,000 cd	9933 ab	2252 bc	4432 bc	251 b	24.8 ab	22,591 c–g	96 a–c	29 ab	6.6 b
KfoA	3.75ab	4871 b–e	48,708 a–c	11,284 ab	2461 a–c	5568 ab	444 b	29.7 a	29,705 a–e	146 a	32 ab	6.9 b
KfoB	3.41 a–d	5138 b–e	43,713 b–d	10,310 ab	2306 bc	5351 a–c	247 b	28.7 ab	29,143 a–e	127 a–c	33 ab	6.5 b
Nco	4.22 ab	6683 ab	62,969 a	13,970 a	2809 a	4912 bc	1523 a	27.5 ab	32,065 a–c	143 ab	40 ab	5.3 b
Annapolis	4.05 ab	5432 a–e	44,883 b–d	10,575 ab	2305 bc	4080 bc	357 b	27.7 ab	18,070 fg	87 a–c	31 ab	8.0 ab
Darkland	2.79 cd	4107 de	31,240 d	7139 b	1764 d	2887 c	217 b	20.7 b	18,428 fg	88 a–c	23 b	3.8 b
Eruption	3.89 ab	4724 c–e	55,544 ab	9693 ab	2112 b–d	3778 bc	402 b	27.4 ab	16,679 g	101 a–c	43 a	12.4 a
Firecracker	4.43 a	7266 a	50,426 a–c	7849 b	2320 bc	3493 bc	216 b	29.9 a	20,199 d–g	70 bc	41 a	6.9 b
Grand Rapids	3.76 ab	5139 b–e	45,783 b–d	9212 ab	2318 bc	3377 bc	253 b	24.9 ab	25,025 c–g	95 a–c	34 ab	6.0 b
Merlot	3.72 ab	6375 a–c	43,438 b–d	7627 b	2063 cd	3253 bc	267 b	26.7 ab	19,831 e–g	67 c	38 ab	7.7 b
SM13-L2	2.52 d	4121 de	33,019 d	9119 ab	2075 b–d	3740 bc	181 b	24.5 ab	27,671 b–f	97 a–c	28 ab	5.5 b
**Accession**	**Mo**	**Ni**	**Na**	**Si**	**Al**	**Ba**	**Cd**	**Cr**	**Li**	**Sr**	**Ti**	
RSL-NAR	0.48 b	1.13 b–d	6072 bc	451 bc	175 b	6.4 bc	0.75 a–c	0.48 b	1.40 b	58 ab	11 b	
RSL-NBR	0.70 b	0.85 cd	12,216 a	438 bc	271 b	5.2 bc	0.77 a–c	0.65 b	1.41 b	53 b	17 b	
RSL-NFR	0.49 b	1.06 b–d	6212 bc	475 bc	161 b	6.6 bc	0.57 bc	0.48 b	1.22 b	48 b	9 b	
GSL	0.45 b	0.84 cd	4089 bc	418 bc	225 b	4.4 c	0.57 bc	0.46 b	0.94 b	43 b	13 b	
GSL-DG	0.63 b	0.89 b–d	6046 bc	472 bc	226 b	5.9 bc	0.65 a–c	0.51 b	0.96 b	52 b	13 b	
KfoA	0.98 b	0.93 b–d	6168 bc	727 b	396 b	5.9 bc	0.96 a–c	0.76 b	1.67 b	55 ab	27 b	
KfoB	0.43 b	0.81 cd	6403 bc	573 bc	210 b	4.9 bc	1.11 ab	0.53 b	1.26 b	50 b	13 b	
Nco	4.04 a	2.18 a	6124 bc	1320 a	1450 a	16.9 a	1.15 a	2.27 a	5.43 a	84 a	99 a	
Annapolis	0.99 b	1.07 b–d	5293 bc	588 bc	328 b	8.2 b	0.88 a–c	0.68 b	1.29 b	57 ab	20 b	
Darkland	0.78 b	0.67 d	2768 c	438 bc	228 b	4.5 c	0.44 c	0.47 b	0.71 b	30 b	13 b	
Eruption	1.06 b	0.93 b–d	6072 bc	543 bc	390 b	6.8 bc	1.17 a	0.75 b	1.36 b	52 b	24 b	
Firecracker	0.54 b	1.65 ab	3966 bc	450 bc	180 b	5.1 bc	0.51 c	0.51 b	0.83 b	39 b	9 b	
Grand Rapids	0.51 b	0.86 b–d	3537 c	318 c	249 b	4.8 bc	0.57 bc	0.53 b	0.86 b	44 b	14 b	
Merlot	0.60 b	1.48 a–c	4959 bc	392 bc	246 b	5.2 bc	0.51 c	0.57 b	0.78 b	37 b	14 b	
SM13-L2	0.24 b	0.55 d	7904 b	289 c	160 b	4.6 c	0.70 a–c	0.37 b	1.01 b	45 b	9 b	

All concentrations are reported in µg/g of dry weight except for N, which is reported as percentage of dry weight. Mean values within a column followed by different letters are significantly different at *p* ≤ 0.05 using Tukey’s HSD test. A range of letters, e.g., “a–c” indicates the first and the last letter of the range in alphabetic order.

## Data Availability

The authors confirm that the data supporting the findings of this study are available within the article and its Supplementary Materials. The original values obtained from the measuring instruments can be provided upon request from the corresponding author.

## Supplementary Materials

The following supporting information can be downloaded at: https://www.mdpi.com/2223-7747/12/19/3467/s1, Figure S1: Phenotypes of lettuce accessions grown under field conditions; Figure S2: Phytochemical accumulation of four different flavonoids from 15 lettuce accessions; Table S1: Effect size (ω^2^) and its significance estimated for the main factors (accessions and experiments) from ANOVA; Table S2: Mean values from individual years for the traits evaluated in more than one experiment; Table S3: Weather data for 2019 to 2022 growing seasons.

## References

[1] World Health Organization Cardiovascular Diseases (CVDs). https://www.who.int/news-room/fact-sheets/detail/cardiovascular-diseases-(cvds).

[2] Kim Y., Je Y. (2017). Flavonoid intake and mortality from cardiovascular disease and all causes: A meta-analysis of prospective cohort studies. Clin. Nutr. ESPEN.

[3] Zhan J., Liu Y.-J., Cai L.-B., Xu F.-R., Xie T., He Q.-Q. (2017). Fruit and vegetable consumption and risk of cardiovascular disease: A meta-analysis of prospective cohort studies. Crit. Rev. Food Sci. Nutr..

[4] Reygaert W.C. (2017). An update on the health benefits of green tea. Beverages.

[5] Basu A., Betts N.M., Mulugeta A., Tong C., Newman E., Lyons T.J. (2013). Green tea supplementation increases glutathione and plasma antioxidant capacity in adults with the metabolic syndrome. Nutr. Res..

[6] Jan A.T., Kamli M.R., Murtaza I., Singh J.B., Ali A., Haq Q.M.R. (2010). Dietary Flavonoid Quercetin and Associated Health Benefits—An Overview. Food Rev. Int..

[7] Hertog M.G.L., Feskens E.J.M., Kromhout D., Hollman P.C.H., Katan M.B. (1993). Dietary antioxidant flavonoids and risk of coronary heart disease: The Zutphen Elderly Study. Lancet.

[8] Weber C., Davis W.V., Lucier G., Wechsler S., Davis W.V., Weber C., Wechsler S., Lucier G., Soria Rodriguez M., Yeh A., Fan X. (2023). Lettuce Trends: Conventional, Organic Growth, and Production. Vegetables and Pulses Outlook.

[9] Simko I., Hayes R.J., Mou B., McCreight J.D., Smith S., Diers B., Specht J., Carver B. (2014). Lettuce and Spinach. Yield Gains in Major U.S. Field Crops.

[10] Hayes R.J., Simko I. (2016). Breeding lettuce for improved fresh-cut processing. Acta Hortic..

[11] Hayes R.J., Sandoya G., Mou B., Simko I., Subbarao K.V. (2018). Release of three iceberg lettuce populations with combined resistance to two soilborne diseases. HortScience.

[12] Damerum A., Chapman M.A., Taylor G. (2020). Innovative breeding technologies in lettuce for improved post-harvest quality. Postharvest Biol. Technol..

[13] Armas Gutierrez I. (2015). Nutritional Enhancement of Lettuce Using Mutational Breeding. Master’s Thesis.

[14] Damerum A., Selmes S.L., Biggi G.F., Clarkson G.J., Rothwell S.D., Truco M.J., Michelmore R.W., Hancock R.D., Shellcock C., Chapman M.A. (2015). Elucidating the genetic basis of antioxidant status in lettuce (*Lactuca sativa*). Hortic. Res..

[15] Cheng D.M., Pogrebnyak N., Kuhn P., Krueger C.G., Johnson W.D., Raskin I. (2014). Development and phytochemical characterization of high polyphenol red lettuce with anti-diabetic properties. PLoS ONE.

[16] Gurdon C., Poulev A., Armas I., Satorov S., Tsai M., Raskin I. (2019). Genetic and Phytochemical Characterization of Lettuce Flavonoid Biosynthesis Mutants. Sci. Rep..

[17] Gurdon C., Kozik A., Tao R., Poulev A., Armas I., Michelmore R.W., Raskin I. (2021). Isolating an active and inactive CACTA transposon from lettuce color mutants and characterizing their family. Plant Physiol..

[18] Kim M.J., Moon Y., Tou J.C., Mou B., Waterland N.L. (2016). Nutritional value, bioactive compounds and health benefits of lettuce (*Lactuca sativa* L.). J. Food Compos. Anal..

[19] Simko I. (2019). Genetic variation and relationship among content of vitamins, pigments, and sugars in baby leaf lettuce. Food Sci. Nutr..

[20] Ferreres F., Gil M.I., Castañer M., Tomás-Barberán F.A. (1997). Phenolic Metabolites in Red Pigmented Lettuce (*Lactuca sativa*). Changes with Minimal Processing and Cold Storage. J. Agric. Food Chem..

[21] Llorach R., Martínez-Sánchez A., Tomás-Barberán F.A., Gil M.I., Ferreres F. (2008). Characterisation of polyphenols and antioxidant properties of five lettuce varieties and escarole. Food Chem..

[22] Medina-Lozano I., Bertolín J.R., Díaz A. (2021). Nutritional value of commercial and traditional lettuce (*Lactuca sativa* L.) and wild relatives: Vitamin C and anthocyanin content. Food Chem..

[23] Cheng D.M., Pogrebnyak N., Kuhn P., Poulev A., Waterman C., Rojas-Silva P., Johnson W.D., Raskin I. (2014). Polyphenol-rich Rutgers Scarlet Lettuce improves glucose metabolism and liver lipid accumulation in diet-induced obese C57BL/6 mice. Nutrition.

[24] Cheng D.M., Roopchand D.E., Poulev A., Kuhn P., Armas I., Johnson W.D., Oren A., Ribnicky D., Zelzion E., Bhattacharya D. (2016). High phenolics Rutgers Scarlet Lettuce improves glucose metabolism in high fat diet-induced obese mice. Mol. Nutr. Food Res..

[25] Calderón-Montaño J.M., Burgos-Morón E., Pérez-Guerrero C., López-Lázaro M. (2011). A review on the dietary flavonoid kaempferol. Mini. Rev. Med. Chem..

[26] Hertog M.G.L., Hollman P.C.H., Katan M.B. (1992). Content of potentially anticarcinogenic flavonoids of 28 vegetables and 9 fruits commonly consumed in the Netherlands. J. Agric. Food Chem..

[27] Justesen U., Knuthsen P., Leth T. (1998). Quantitative analysis of flavonols, flavones, and flavanones in fruits, vegetables and beverages by high-performance liquid chromatography with photo-diode array and mass spectrometric detection. J. Chromatogr. A.

[28] Inocencio C., Rivera D., Alcaraz F., Tomás-Barberán F.A. (2000). Flavonoid content of commercial capers (*Capparis spinosa*, *C. sicula* and *C. orientalis*) produced in mediterranean countries. Eur. Food Res. Technol..

[29] Franke A.A., Custer L.J., Arakaki C., Murphy S.P. (2004). Vitamin C and flavonoid levels of fruits and vegetables consumed in Hawaii. J. Food Compos. Anal..

[30] Yang R.Y., Lin S., Kuo G. (2008). Content and distribution of flavonoids among 91 edible plant species. Asia Pac. J. Clin. Nutr..

[31] Horiba T., Nishimura I., Nakai Y., Abe K., Sato R. (2010). Naringenin chalcone improves adipocyte functions by enhancing adiponectin production. Mol. Cell Endocrinol..

[32] Iwamura C., Shinoda K., Yoshimura M., Watanabe Y., Obata A., Nakayama T. (2010). Naringenin Chalcone Suppresses Allergic Asthma by Inhibiting the Type-2 Function of CD4 T Cells. Allergol. Int..

[33] Yamamoto T., Yoshimura M., Yamaguchi F., Kouchi T., Tsuji R., Saito M., Obata A., Kikuchi M. (2004). Anti-allergic Activity of Naringenin Chalcone from a Tomato Skin Extract. Biosci. Biotechnol. Biochem..

[34] Muir S.R., Collins G.J., Robinson S., Hughes S., Bovy A., Ric De Vos C.H., van Tunen A.J., Verhoeyen M.E. (2001). Overexpression of petunia chalcone isomerase in tomato results in fruit containing increased levels of flavonols. Nat. Biotechnol..

[35] Saito K., Yonekura-Sakakibara K., Nakabayashi R., Higashi Y., Yamazaki M., Tohge T., Fernie A.R. (2013). The flavonoid biosynthetic pathway in Arabidopsis: Structural and genetic diversity. Plant Physiol. Biochem..

[36] Li Q., Kubota C. (2009). Effects of supplemental light quality on growth and phytochemicals of baby leaf lettuce. Environ. Exp. Bot..

[37] Goto E., Hayashi K., Furuyama S., Hikosaka S., Ishigami Y. (2016). Effect of UV light on phytochemical accumulation and expression of anthocyanin biosynthesis genes in red leaf lettuce. Acta Hortic..

[38] Kitazaki K., Fukushima A., Nakabayashi R., Okazaki Y., Kobayashi M., Mori T., Nishizawa T., Reyes-Chin-Wo S., Michelmore R.W., Saito K. (2018). Metabolic Reprogramming in Leaf Lettuce Grown Under Different Light Quality and Intensity Conditions Using Narrow-Band LEDs. Sci. Rep..

[39] Meng Q., Boldt J., Runkle E.S. (2020). Blue Radiation Interacts with Green Radiation to Influence Growth and Predominantly Controls Quality Attributes of Lettuce. J. Amer. Soc. Hort. Sci..

[40] Meng Q., Runkle E.S. (2020). Growth Responses of Red-Leaf Lettuce to Temporal Spectral Changes. Front. Plant Sci..

[41] Sytar O., Zivcak M., Bruckova K., Brestic M., Hemmerich I., Rauh C., Simko I. (2018). Shift in accumulation of flavonoids and phenolic acids in lettuce attributable to changes in ultraviolet radiation and temperature. Sci. Hortic..

[42] Ryan K.G., Swinny E.E., Winefield C., Markham K.R. (2001). Flavonoids and UV Photoprotection in Arabidopsis Mutants. Z. aturforsch C. J. Biosci..

[43] Havaux M., Kloppstech K. (2001). The protective functions of carotenoid and flavonoid pigments against excess visible radiation at chilling temperature investigated in *Arabidopsis npq* and *tt* mutants. Planta.

[44] Landry L.G., Chapple C.C., Last R.L. (1995). Arabidopsis mutants lacking phenolic sunscreens exhibit enhanced ultraviolet-B injury and oxidative damage. Plant Physiol..

[45] Gazula A., Kleinhenz M.D., Scheerens J.C., Ling P.P. (2007). Anthocyanin levels in nine lettuce (*Lactuca sativa*) cultivars: Influence of planting date and relations among analytic, instrumented, and visual assessments of color. HortScience.

[46] Marin A., Ferreres F., Barberá G.G., Gil M.I. (2015). Weather variability influences color and phenolic content of pigmented baby leaf lettuces throughout the season. J. Agric. Food Chem..

[47] Cao J., Chen W., Zhang Y., Zhang Y., Zhao X. (2010). Content of Selected Flavonoids in 100 Edible Vegetables and Fruits. Food Sci. Technol. Res..

[48] Arabbi P.R., Genovese M.I., Lajolo F.M. (2004). Flavonoids in Vegetable Foods Commonly Consumed in Brazil and Estimated Ingestion by the Brazilian Population. J. Agric. Food Chem..

[49] Jaakola L., Hohtola A. (2010). Effect of latitude on flavonoid biosynthesis in plants. Plant Cell Environ..

[50] Zahra N., Hafeez M.B., Shaukat K., Wahid A., Hasanuzzaman M. (2021). Fe toxicity in plants: Impacts and remediation. Physiol. Plant..

[51] Sthapit Kandel J., Peng H., Hayes R.J., Mou B., Simko I. (2020). Genome-wide association mapping reveals loci for shelf life and developmental rate of lettuce. Theor. Appl. Genet..

[52] Rosental L., Still D.W., You Y., Hayes R.J., Simko I. (2021). Mapping and identification of genetic loci affecting earliness of bolting and flowering in lettuce. Theor. Appl. Genet..

[53] Padmavati M., Reddy A.R. (1999). Flavonoid Biosynthetic Pathway and Cereal Defence Response: An Emerging Trend in Crop Biotechnology. J. Plant Biochem. Biotechnol..

[54] Ramaroson M.-L., Koutouan C., Helesbeux J.-J., Le Clerc V., Hamama L., Geoffriau E., Briard M. (2022). Role of Phenylpropanoids and Flavonoids in Plant Resistance to Pests and Diseases. Molecules.

[55] Simko I., Hayes R.J., Kramer M. (2012). Computing Integrated Ratings from Heterogeneous Phenotypic Assessments: A Case Study of Lettuce Postharvest Quality and Downy Mildew Resistance. Crop Sci..

[56] Simko I., Peng H., Sthapit Kandel J., Zhao R. (2022). Genome-wide association mapping reveals genomic regions frequently associated with lettuce field resistance to downy mildew. Theor. Appl. Genet..

[57] Simko I., Hayes R.J., Bull C.T., Mou B., Luo Y., Trent M.A., Atallah A.J., Ryder E.J., Sideman R.G. (2014). Characterization and Performance of 16 New Inbred Lines of Lettuce. HortScience.

[58] Hayes R.J., Wu B., Pryor B., Chitrampalam P., Subbarao K. (2010). Assessment of Resistance in Lettuce (*Lactuca sativa* L.) to Mycelial and Ascospore Infection by *Sclerotinia minor* Jagger and *S. sclerotiorum* (Lib.) de Bary. HortScience.

[59] Simko I., Sthapit Kandel J., Peng H., Zhao R., Subbarao K.V. (2023). Genetic determinants of lettuce resistance to drop caused by Sclerotinia minor identified through genome-wide association mapping frequently co-locate with loci regulating anthocyanin content. Theor. Appl. Genet..

[60] Mamo B.E., Hayes R.J., Truco M.J., Puri K.D., Michelmore R.W., Subbarao K.V., Simko I. (2019). The genetics of resistance to lettuce drop (*Sclerotinia* spp.) in lettuce in a recombinant inbred line population from Reine des Glaces × Eruption. Theor. Appl. Genet..

[61] Hasegawa D.K., Del Pozo-Valdivia A.I. (2022). Epidemiology and Economic Impact of Impatiens Necrotic Spot Virus: A Resurging Pathogen Affecting Lettuce in the Salinas Valley of California. Plant Dis..

[62] Simko I., Richardson C.E., Wintermantel W.M. (2017). Variation within *Lactuca* spp. for Resistance to Impatiens necrotic spot virus. Plant Dis..

[63] Simko I., Hasegawa D.K., Peng H., Zhao R. (2023). Genetic and physiological determinants of lettuce partial resistance to Impatiens necrotic spot virus. Front. Plant Sci..

[64] Peng H., Simko I. (2023). Extending lettuce shelf life through integrated technologies. Curr. Opin. Biotechnol..

[65] Van Dun C.M.P. (2014). Screening Method for Selecting Plants that Show Reduced Wound-Induced Surface Discolouration and Plant and Plant Parts thus Obtained. Patent US.

[66] Simko I., Atallah A.J., Ochoa O.E., Antonise R., Galeano C.H., Truco M.J., Michelmore R.W. (2013). Identification of QTLs conferring resistance to downy mildew in legacy cultivars of lettuce. Sci. Rep..

[67] Subbarao K.V. (1998). Progress toward integrated management of lettuce drop. Plant Dis..

[68] Parry C., Blonquist J.M., Bugbee B. (2014). In situ measurement of leaf chlorophyll concentration: Analysis of the optical/absolute relationship. Plan Cell Environ..

[69] van den Berg A.K., Perkins T.D. (2005). Nondestructive estimation of anthocyanin content in autumn sugar maple leaves. HortScience.

[70] Simko I. (2020). Predictive modeling of a leaf conceptual midpoint quasi-color (CMQ) using an artificial neural network. Sensors.

[71] Hayes R.J., Galeano C.H., Luo Y., Antonise R., Simko I. (2014). Inheritance of decay of fresh-cut lettuce in a recombinant inbred line population from ‘Salinas 88’ × ‘La Brillante’. J. Am. Soc. Hortic. Sci..

[72] Simko I., Hayes R.J. (2018). Accuracy, reliability, and timing of visual evaluations of decay in fresh-cut lettuce. PLoS ONE.

[73] Rueden C.T., Schindelin J., Hiner M.C., DeZonia B.E., Walter A.E., Arena E.T., Eliceiri K.W. (2017). ImageJ2: ImageJ for the next generation of scientific image data. BMC Bioinform..

[74] Rossini Oliva S., Raitio H., Mingorance M.D. (2003). Comparison of two wet digestion procedures for multi-element analysis of plant samples. Commun. Soil Sci. Plant Anal..

[75] United States Environmental Protection Agency (EPA) Method 6010B. Inductively Coupled Plasma-Atomic Emission Spectrometry. https://www.epa.gov/sites/default/files/documents/6010b.pdf.

[76] Soltanpour P.N., Johnson G.W., Workman S.M., Jones J.B., Miller R.O., Sparks D.L., Page A.L., Helmke P.A., Loeppert R.H., Soltanpour P.N., Tabatabai M.A., Johnston C.T., Sumner M.E. (1996). Inductively coupled plasma emission spectrometry and inductively coupled plasma-mass spectrometry. Methods of Soil Analysis: Part 3 Chemical Methods.

[77] Bremner J.M., Sparks D.L., Page A.L., Helmke P.A., Loeppert R.H., Soltanpour P.N., Tabatabai M.A., Johnston C.T., Sumner M.E. (1996). Nitrogen-total. Methods of Soil Analysis: Part 3 Chemical Methods.

