# Supplementary material for: Phytochemical and Agronomic Characterization of High-Flavonoid Lettuce Lines Grown under Field Conditions
Source: Plants (Basel). 2023 Oct 2;12(19):3467. doi: 10.3390/plants12193467 (PMC10574981; doi:10.3390/plants12193467)
Supplement: Supplementary file 1 [file plants-12-03467-s001.zip › plants-2627692-supplementary.pdf]

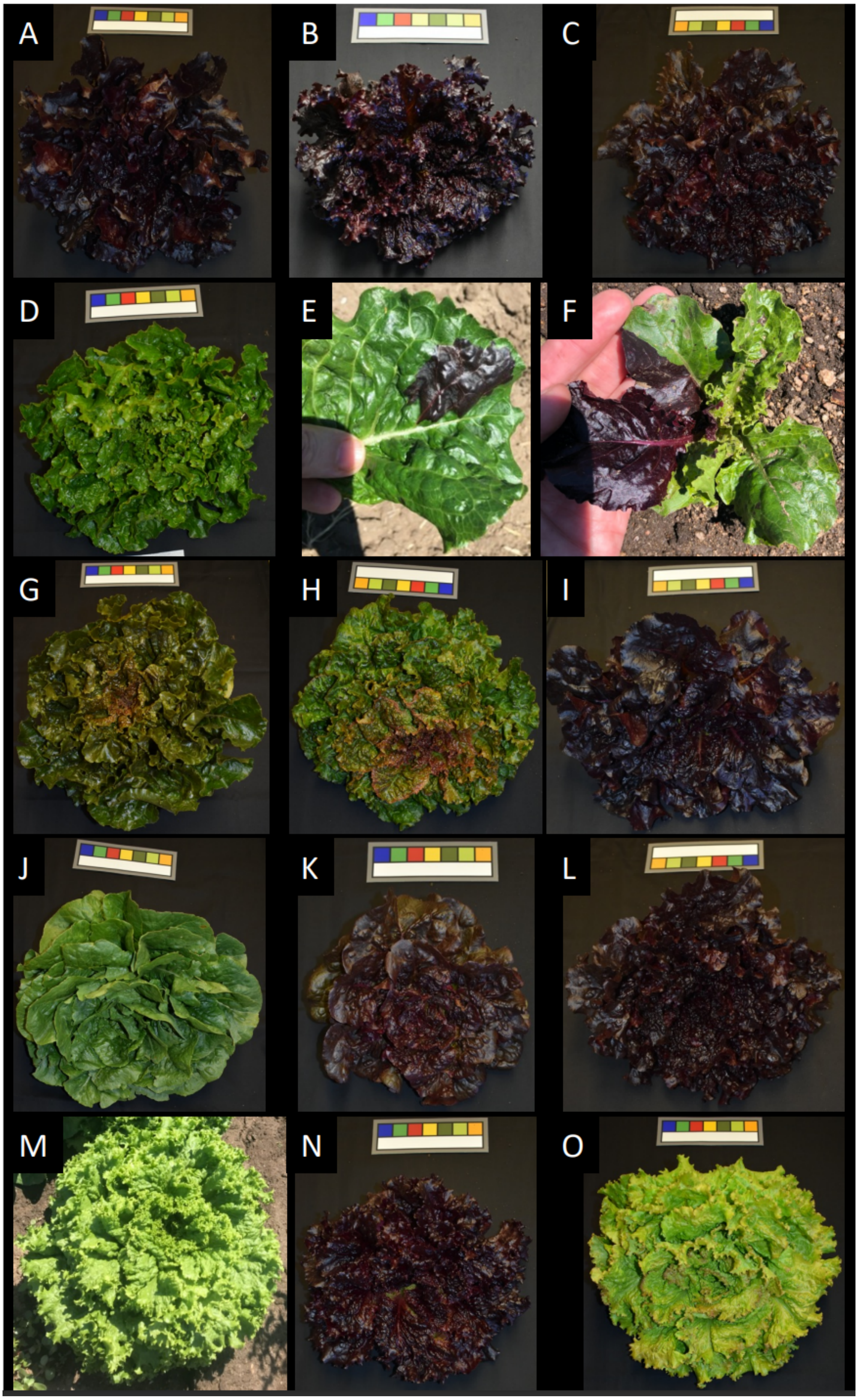

Figure S1. Phenotypes of lettuce accessions grown under field conditions. RSL-NAR (A), RSL-NBR (B), RSL-NFR (C), GSL (D), occasional red patches (E) or even whole leaves on GSL (F), GSL-DG (G), KfoB (H), Annapolis (I), Darkland (J), Eruption (K), Firecracker (L), Grand Rapids (M), Merlot (N), and SM13-L2 (O).

SUPPLEMENTARY MATERIALS

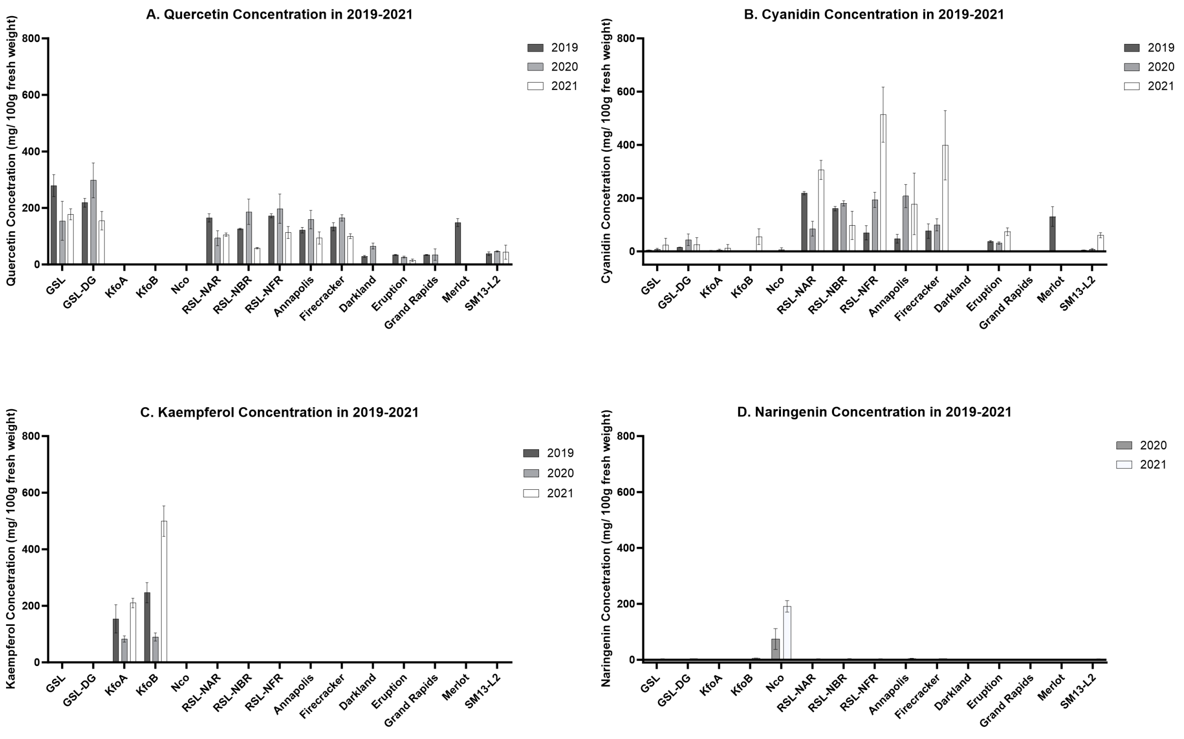

Figure S2. Phytochemical accumulation of four different flavonoids from 15 lettuce accessions using UPLC-MS/MS over the span of 2019-2021, n = 4 for 2019 measurements and n = 3 for 2020 and 2021 measurements. (A) Average quercetin content in mg/100 g fresh weight. (B) Average cyanidin content in mg/100 g fresh weight. (C) Average kaempferol content in mg/100 g fresh weight. (D) Average naringenin content (converted from naringenin chalcone during extraction) in mg/100 g fresh weight.

Table S1. Effect size (ω^2) and its significance estimated for the main factors (accessions and experiments) from ANOVA.

Trait	No. of experiments	Accession effect		Experiment Effect	
		ω^2	LogWorth	ω^2	LogWorth
**Plant biomass production**					
Plant height	1	0.84	64.5	-	-
Plant width	1	0.73	44.7	-	-
Plant weight	2	0.47	11.7	0.31	13.4
**Pigments**					
Anthocyanins	2	0.92	28.8	0.02	4.3
Chlorophyll	2	0.37	4.6	0.06	2.4
**Disease resistance**					
Downy mildew	2	0.02	0.4	0.00	0.5
INSV	3	0.08	1.5	0.13	4.7
Lettuce drop	2	0.30	6.0	0.01	1.0
**Postharvest quality**					
Deterioration	1	0.44	2.4	-	-
Discoloration	1	0.71	11.7	-	-
**Flavonoids**					
Cyanidin	3	0.41	10.7	0.09	5.0
Kaempferol	3	0.67	24.1	0.03	2.9
Naringenin	2	0.88	29.1	0.01	2.1
Quercetin	3	0.70	26.9	0.03	3.8
**Mineral composition**					
Aluminum	1	0.91	13.5	-	-
Barium	1	0.86	10.9	-	-
Boron	1	0.28	1.5	-	-
Cadmium	1	0.57	4.2	-	-
Calcium	1	0.35	2.0	-	-
Chloride	1	0.80	8.6	-	-
Chromium	1	0.89	12.4	-	-
Copper	1	0.55	3.9	-	-
Iron	1	0.90	12.9	-	-
Lithium	1	0.82	9.3	-	-
Magnesium	1	0.59	4.5	-	-
Manganese	1	0.44	2.8	-	-
Molybdenum	1	0.91	13.4	-	-
Nickel	1	0.68	5.8	-	-
Nitrogen	1	0.69	5.8	-	-
Phosphorus	1	0.67	5.7	-	-
Potassium	1	0.72	6.7	-	-

Silicon	1	0.75	7.3	-	-
Sodium	1	0.67	5.7	-	-
Strontium	1	0.53	3.6	-	-
Sulfur	1	0.68	5.8	-	-
Titanium	1	0.90	13.1	-	-
Zinc	1	0.28	1.5	-	-

Analyses were performed on traits related to biomass production, pigments content, disease resistance, postharvest quality, flavonoids content, and mineral element composition. LogWorth values are logarithmic transformations of p-values (LogWorth = -log10(p-value)), thus LogWorth of 2 is equivalent to p-value of 0.01. When a single experiment was performed for a trait, results are provided for the effect of accessions only.

Table S2. Mean values from individual years for the traits evaluated in more than one experiment.

Accession	Plant weight (g)		Chlorophyll (SPAD-sqrt)		Anthocyanins (ACI-lb)		Downy mildew (rating)		Lettuce drop (incidence %)		INSV (incidence %)		
	2019	2021	2019	2022	2019	2022	2019	2020	2020	2021	2020	2021	2022
RSL-NAR	342	364	6.4	6.6	7.2	8.8	0.01	0.07	64	40	7	4	20
RSL-NBR	152	714	6.6	6.2	6.2	8.8	0.00	0.72	65	45	2	4	2
RSL-NFR	328	540	6.4	6.4	6.6	9.0	0.70	1.66	72	50	7	6	20
GSL	454	688	7.2	6.8	3.4	3.4	0.55	0.16	70	58	0	6	33
GSL-DG	418	602	6.6	6.8	3.9	4.1	1.89	0.14	67	43	21	8	4
KfoA	207	615	6.1	6.1	3.0	3.6	0.03	0.03	83	59	0	1	71
KfoB	430	570	6.0	6.2	5.6	3.8	1.36	0.11	86	58	0	0	30
Nco	-	10	-	5.2	-	1.3	-	-	92	2	-	4	2
Annapolis	303	563	6.5	6.9	6.9	8.9	0.00	1.28	24	42	4	4	7
Darkland	950	981	7.1	7.3	3.3	3.5	1.05	0.53	42	88	26	16	21
Eruption	274	716	6.4	6.4	5.5	5.9	0.00	1.70	13	25	4	5	4
Firecracker	305	473	5.9	6.9	7.0	7.2	0.00	0.80	79	57	15	2	16
Grand Rapids	305	589	4.7	7.1	1.3	2.5	0.03	0.03	44	100	14	10	12
Merlot	244	627	5.8	6.6	6.8	8.6	0.10	0.10	58	66	8	1	37
SM13-L2	486	1042	4.1	5.9	1.7	1.9	0.10	0.10	50	58	8	3	13

Missing values are indicated by dashes ‘-‘.

Table S3. Weather data for 2019 to 2022 growing seasons.

Growing season (Year)	Total ETo (mm)	Total Precip (mm)	Avg Sol Rad (W/m^2)	Avg Vap Pres (kPa)	Avg Max Air Temp (°C)	Avg Min Air Temp (°C)	Avg Air Temp (°C)	Avg Max Rel Hum (%)	Avg Min Rel Hum (%)	Avg Rel Hum (%)	Avg Dew Point (°C)	Avg Wind Speed (m/s)	Avg Soil Temp (°C)
2019	419	38.9	289	1.4	21.2	11.9	15.5	94.0	61.0	79.3	11.9	2.0	20.5
2020	507	5.8	306	1.4	23.3	12.0	16.5	95.7	54.7	75.3	12.0	4.1	18.7
2021	491	1.3	312	1.4	21.7	11.6	15.5	96.3	60.0	79.7	12.0	4.1	19.2
2022	528	0.9	321	1.3	22.3	10.6	15.4	91.7	52.3	73.0	10.5	4.3	20.6

Data were obtained from weather station #214 at https://cimis.water.ca.gov
